# When money talks: Judging risk and coercion in high-paying clinical trials

**DOI:** 10.1371/journal.pone.0227898

**Published:** 2020-01-31

**Authors:** Christina Leuker, Lasare Samartzidis, Ralph Hertwig, Timothy J. Pleskac

**Affiliations:** 1 Center for Adaptive Rationality, Max Planck Institute for Human Development, Berlin, Germany; 2 Department of Psychology, The University of Kansas, Lawrence, Kansas, United States of America; University of Florida, UNITED STATES

## Abstract

Millions of volunteers take part in clinical trials every year. This is unsurprising, given that clinical trials are often much more lucrative than other types of unskilled work. When clinical trials offer very high pay, however, some people consider them repugnant. To understand why, we asked 1,428 respondents to evaluate a hypothetical medical trial for a new Ebola vaccine offering three different payment amounts. Some respondents (27%) used very high pay (*£*10,000) as a cue to infer the potential risks the clinical trial posed. These respondents were also concerned that offering *£*10,000 was coercive— simply too profitable to pass up. Both perceived risk and coercion in high-paying clinical trials shape how people evaluate these trials. This result was robust within and between respondents. The link between risk and repugnance may generalize to other markets in which parties are partially remunerated for the risk they take and contributes to a more complete understanding of why some market transactions appear repugnant.

## Introduction

All of us, with the exception of the independently wealthy and the unemployed, take money for the use of our body. (Nussbaum, 1998, p. 693) [1]

In 2015, 128 people took part in a drug study that offered €1,900, travel expenses, and a two-week stay at a pharmaceutical research institute in Rennes in return for swallowing a drug on 10 consecutive days, undergoing extensive medical tests, and providing at least 40 blood samples. Later, news broke that one volunteer died and six others were hospitalized. Experts underlined “the astonishing and unprecedented nature” of these incidents [2]. This case, while extreme, illustrates that despite prior safeguards such as pretests in animals it is not possible to fully anticipate the risks [3]. Does this ambiguity impact how third party observers evaluate clinical trials? Why was the payment frequently considered to be too high in the wake of the tragedy [4]?

These are important questions to ask given the role clinical trials play in the development of new medical treatments. The transition from animal testing to phase 1 trials, which assess the safety and effectiveness of a particular substance in humans, is critical. Millions of volunteers, both healthy and with existing health conditions, are sought for clinical trials every year. While some volunteers with existing conditions may take part in medical research studies in the hopes of improving their health, for healthy volunteers financial incentives are the primary motivator to participate [5, 6]. The standard procedure for clinical trial volunteers is to sign an informed consent that outlines the details of the study including potential risks, and the research institute and the people offering their body engage in a voluntary market transaction. This transaction is, to some people, no different from any other market transaction [7].

To other people, however, clinical trials are hardly comparable to market transactions such as getting a haircut. Instead they deem them as repugnant [8]. That is, clinical trials are sometimes considered so morally repulsive, distasteful, or inappropriate that third parties want to prevent them [9]. Thereby, they fall into a similar category as pornography, surrogate motherhood or paid kidney donations [8]. All of these transactions commodify the human body—although clearly not all of these transactions may be considered equally repugnant, and some people or cultures do not see them as repugnant at all. The repugnance of clinical trials has so far mainly been attributed to payment coercing or unduly influencing volunteers [9, 10]. Note that the terms “undue influence” and “coercion” are often used interchangeably, but see [4], 2017 for a disambiguation. Coercion may arise because payment in clinical trials can be much higher than in other types of unskilled work for which the default payment is the minimum wage. For instance, in 2015, minimum wage workers in France would have earned €729 in two weeks, less than 40% of what the 128 volunteers in the drug study earned in Rennes [11].

In an experimental test of the perceived ethicality of clinical trials in the eyes of third parties, [9] found that some people, whom we call “doubtful respondents”, rated a clinical trial offering $10,000 as more coercive than the same trial offering $1,000. Note that we call the “ethicists” of the Ambuehl et al. (2015) survey “doubtful respondents” and refer to “economists” of the same survey as “trustful respondents” to reflect the fact that doubtful respondents are consistently more critical than trustful respondents of high-paying clinical trials; and trustful respondents do not consider the research institute to have any ulterior motives when offering high pay (e.g. to plan and conduct a trial that exposes participants to relatively high risks; or coerces them to participate). Consistent with the evaluation that high-paying clinical trials can be coercive, doubtful respondents also thought participants would regret enrolling in the higher paying study and that they would be better off not participating. Finally, the doubtful respondents stated they would be less likely to approve such a study if they were on an institutional review board (IRB) panel. A second group of respondents, whom we call “trustful respondents”, rated the trial offering $10,000 as less coercive. They thought that people would be better off if they took part and that they would not regret participating. Trustful respondents on an imagined IRB panel were more likely to approve the study offering $10,000 than the one offering $1,000.

The sense of possible coercion may not be the only factor that discerns between these two groups. One key difference between clinical trial participation and many other types of job that commodify the human body is that clinical trials expose participants to unknown health risks [3]. Clinical trial volunteers may be compensated after side effects are experienced, but policies vary: Member states of the European Union must offer systematic compensation for research-related injuries; no such regulation exists in the United States [12]. In some cases, the compensation volunteers receive for participating in a clinical trial may even be considered to partially offset the suspected risks to which they are exposed: Around one third of surveyed research institutes reported that one rule of thumb for determining pay is the anticipated risk participants incur [13]. Such a practice is highly controversial, but not explicitly prohibited [4], and it may explain why payment is sometimes treated as a cue indicating the risk of negative consequences from participating in a clinical trial ([14]; [4]; though results are inconsistent; see [15]). Research institutes also reported that participants are paid to compensate them for their time (87%), inconvenience (84%), or travel (68%) [13]. In general, payment ranges are large across different clinical trials [13] and payment is not likely to be a highly valid cue for the underlying risk for at least two reasons: Payments that reflect anticipated risks are not consistently employed as a rule and the precise risks of a clinical trial are unknown a priori.

Nevertheless, third parties to the market transaction (i.e, are neither volunteers nor providers of clinical trials) who judge the ethicality—and by extension, the repugnance—of a high-paying clinical trial may not only take into account the coercion caused by the high payoffs, but also the risks that the payoff signals. Very high payoffs offered in clinical trials could highlight the potential harm to participants and thereby decrease third party’s approval of the study. In addition, it is also conceivable that trials subjectively perceived to be riskier have, for whatever reason, particularly coercive qualities. In both cases, the difference between the two types of responses in [9] may at least partly be due to different inferences about the implied risks. This is not an uncontested hypothesis. Ambuehl et al. (2015) maintained that the risks of the $1,000 and $10,000 payment schemes were seen as equal because respondents got the same description of the trial and saw all the possible payoff schemes (see their Footnote 7). Moreover, others have argued that high payment amounts may conceal the risk involved in taking part in a study [3, 16], or even compromise prospective participants’ ability to think carefully about the risks and benefits involved [17].

To investigate whether the judged ethicality of high-paying clinical trials is influenced by the risks inferred from the payoff magnitude, we put online survey respondents in the shoes of an IRB board—as a third party evaluator—and presented them with a hypothetical medical trial that compensated volunteers with *£*50, *£*1,000, or *£*10,000 (materials adapted and extended from Ambuehl et al., 2015). Respondents estimated how many prospective participants would experience side effects, and evaluated the clinical trial on several other dimensions pertaining to coercion and ethicality. Each respondent saw all three payment amounts, but our focus is on responses to the first payment amount a respondent saw. Each respondent therefore appears only once in the analyses. Within-respondent analyses are consistent, and shown in the S1 Supplementary materials.

We addressed five research questions, of which questions 2–4 were not previously investigated empirically:

1. Do the results from Ambuehl et al. (2015) replicate?
2. Do people who evaluate a clinical trial perceive higher payment to be associated with higher risk?
3. Why do people consider high pay in clinical trials to be ethically inappropriate—coercion, (subjective) risk, or both?
4. How do payment-dependent inferences shape judgments on the repugnance of clinical trials?
5. What dissociates between doubtful and trustful respondents (exploratory)?

We preregistered our analytic approach (osf.io/kumge). We defined a successful replication as an interaction effect between the respondent types “doubtful” and “trustful” (initially referred to as “ethicists” and “economists”) and payoff amount on IRB approval. For trustful respondents, IRB approval should increase, when payoff increases from *£*1000 to *£*10,000. For doubtful respondents, IRB approval should decrease, when payoff increases from *£*1000 to *£*10,000. In addition, we predicted that respondents in the *£*10,000 condition infer more volunteers to experience side effects compared to the *£*1000 condition, and that the ethicality judgments depend on inferred side effects. Data and analysis code are posted on the Open Science Framework (osf.io/5kewt/).

## Materials and methods

### Participants

In total, *N* = 1,565 respondents completed our survey posted on Prolific Academic for a flat payment of *£*2.10. Inclusion criteria were fluency in English (self-assessed) and a minimum approval rate of 80% in earlier studies completed on the platform. The data were collected in two waves (*N* = 354 in wave 1, *N* = 1, 211 in wave 2). The first sample size was determined based on the availability, and a reasonable allocation, of funds (osf.io/yftvh), since our survey added several sets of questions that lengthened the original 12-minute survey to an estimated 25 minutes. Wave 1 was smaller than the sample size in the original study (*N* = 1, 445, [9]).

After examining the results, we sought additional evidence to test our predictions regarding the estimated side effects *between* respondents. We aimed to recruit an additional *N* = 1, 200 respondents in wave 2 (osf.io/kumge). In wave 2, we also included a novel “repugnance” measure that assessed how ethically appropriate respondents considered clinical trials in general. This measure allowed us to assess individual differences in the degree to which people consider clinical trials to be repugnant.

The total sample size of waves 1 and 2 roughly matched that of the original study. Inclusion criteria, predictions, survey questions, and the reasoning behind collecting additional responses were preregistered. The surveys were approved by the IRB of the Max Planck Institute for Human Development. We collapsed our data in the main manuscript and report effects of wave 1 versus wave 2 in the analyses of interest in the S1 Supplementary materials (in short: They were largely independent of wave). We analyzed data from *N* = 1,428 (*N* = 316 in wave 1, *N* = 1,112 in wave 2) respondents who passed three simple attention checks (questions pertaining to the instructions and the clinical trial description on the same page). The final sample consisted of 885 females, 535 males, and eight who identified as “other,” was on average 36.1 years old (range 18–78 years, *SD* = 11.8), and had a median income of *£*35,000. Further sample characteristics and their distributions are plotted in S5. On average, respondents took 20 minutes to complete the study.

### Survey

#### Vignette

We adopted a vignette describing a hypothetical clinical trial designed to test an Ebola vaccination [9]. The level of detail provided in this vignette is similar to the information available to IRBs and policy makers as they judge whether a trial should be approved—the situation to which we seek to generalize. Respondents were thus put in the position of an IRB panelist evaluating the trial, described as a phase 1 trial which sought to test the vaccination for the first time on 100 female volunteers after pretests in rats and chimps. The vaccine was described as having “low, but nonzero risks” (see S1 Supplementary materials for full vignette). The clinical trial remunerated prospective participants with one of three payment amounts [*£*50/*£*1,000/*£*10,000]. Each respondent saw the vignette with all three payment amounts, but the primary analyses in the manuscript are based on the first payment amount each respondent saw (i.e., between respondents). The first payment amount each respondent saw was randomized. In the final sample, 487 respondents were first presented with the clinical trial offering *£*50, 480 respondents were first presented with the clinical trial offering *£*1,000, and 461 respondents were first presented with the clinical trial offering *£*10,000. Within-respondent analyses were consistent with the between-respondent analyses and are reported in the S1 Supplementary materials. The original survey included U.S. residents recruited via Amazon Mechanical Turk and hence used U.S. currency ($). As our sample was British, we used U.K. currency (*£*). We did not transform the values according to exchange rates. The primary reason for choosing these payment amounts was consistency with the study we sought to replicate [9]. In addition, one study identified *£*75 and *£*10,000 as lower and upper bounds of the payment subjects expect in some types of clinical trials [18].

#### Side effects

After reading the scenario, respondents were asked to assess how many of the 100 participants they expected to experience [any/mild/severe] side effects.

#### Clinical trial evaluations

As in the original survey, each respondent evaluated the clinical trial answering the following questions in order. *(1) IRB approval*. Suppose you are a member of the ethics committee that has to approve the institute’s study with [*payment*]. How would you decide? *(2) Personal approval*. How much do you personally approve of the institute’s proposal to enlist and compensate study participants from both rich and poor neighborhoods in this way? *(3) P (better off without)*. [*description of A.S., a woman earning* $1,500/*month*] Suppose that 10 women similar to A.S. see the institute’s study participation invitation. How many of the 10 would be better off if the institute had never posted the study participation invitation? In the original survey, respondents rated the ethicality of the trial for two women: one earning *£*1,500/month and one earning minimum wage. In our survey, participants only evaluated the trial for a woman earning *£*1,500/month. Since the minimum wage in the United Kingdom is around *£*1,250/month [19], we did not expect results to differ substantially in these two conditions. *(4) P (enroll)*. How many of the 10 do you think will eventually participate in the study in exchange for [*payment*]? *(5) Voluntariness*. If A.S. decides to participate in the study for [*payment*], how would you describe her decision? (Likert scale with extremes labelled *She was coerced* and *Her decision was entirely voluntary*). *(6) P (regret accepting)*. If A.S. decides to participate in the study, how likely is it that she will later regret her decision? *(7) P (regret rejecting)*. If A.S. decides NOT to participate in the study, how likely is it that she will later regret her decision? (Both on a Likert scale with extremes labelled *Extremely unlikely* and *Extremely likely*).

Since *IRB approval* and *personal approval* were highly correlated, we focus on *IRB approval* in the main manuscript. Similarly, since *P (regret accept)* and *P (regret reject)* were highly correlated, we focus on *P (regret accept)* in the main manuscript. Corresponding models for the other variables can be found in the S1 Supplementary materials.

#### Different types of respondents

At the end of the survey, participants rated the appropriateness of different payments again, side by side, on a 7-point Likert scale (“For each of the following ways of compensating study participants, please indicate how ethically appropriate you think it is. Recall that the study tests for effects of a vaccine, and although nobody expects such side effects to occur, if this were known, there would be no need to run a study. There is no special compensation if side effects occur”). That is, here (in contrast to the beginning of the survey), payment amounts were *directly* juxtaposed. This question, as in [9], was later used to categorize respondents into three types (doubtful, trustful, and others): Doubtful respondents strictly preferred offering *£*1,000 over *£*10,000, trustful respondents strictly preferred offering *£*10,000 to *£*1,000, and others considered both payment amounts equally acceptable. These names reflect that doubtful respondents are more critical of clinical trials (in particular those that offer high pay) throughout, whereas the responses of trustful respondents are consistent with payoff-independent clinical trial risk inferences.

#### Repugnance

In our second wave of data collection, we also asked respondents to rate the repugnance of clinical trials in general, based on a novel repugnance measure that consisted of four items: “Clinical trial markets… (a) are deplorable, (b) morally permissible *(R)*, (c) should be banned *(R)*, (d) should be monitored *(R)*”. Participants responded to these questions on a Likert scale from *strongly agree* (1) to *strongly disagree* (7). We recoded the answers to questions 2–4 (R) such that 1 denoted “less repugnant” and 7 denoted “more repugnant”. Based on these items, we computed a repugnance measure for each respondent (their average rating across the four items). Individual responses to questions 1–3 were highly correlated. Responses to question 4 (“should be monitored”) were uncorrelated to the other responses. Since including and excluding question 4 from our repugnance measure led to qualitatively identical results, and we had not predicted it to be uncorrelated to the other measures a priori, we included it in the reported regressions. All repugnance analyses are based on wave 2 data—the repugnance questions had not been included in wave 1 (also see OSF).

#### Numeracy, risk-taking and demographics

Lastly, respondents completed the Berlin Adaptive Numeracy Test [20], an “insurance task” (not reported here, see OSF for wording), and a questionnaire on their self-rated willingness to take risks. Six domain-specific risk-taking items were included (driving, finances, recreation, occupation, health, and social). The wording was as follows: “People can behave differently in different situations. How would you rate your willingness to take risks in [*the financial domain*]?”. A general risk-propensity item was included in wave 2 (“How willing are you to take risks in general?” [21]). Respondents also indicated whether they had ever thought about participating in medical research themselves. The survey concluded with demographic questions (gender, age, education). The final sample, on average, took 22.5 minutes (range 8.7 − 74.5 minutes, *SD* = 10.38) to complete the survey.

### Statistical analyses

We relied on Bayesian estimation techniques [22] and applied Bayesian Generalized Linear Models using Stan in R for regression analyses with the rstanarm package [23]. We ran three chains using Markov Chain Monte Carlo sampler to draw from posterior distributions of parameters, with 10,000–30,000 samples per chain (to ensure an effective sample size of > 10,000 for each coefficient), and a burn-in of 500 samples. We investigated the convergence of posteriors through visual inspection and the Gelman-Rubin statistic [24]. We used weakly informative default priors from the rstanarm package [23]. These are normal distributions centered at 0 for the intercepts and coefficients, with standard deviations scaled to the order of magnitude of the variables; producing wide, conservative priors. In general, we report the mean of the posterior distribution of the parameter or statistic of interest and two-sided 95% equal tail credible intervals (CI) around each value. Our focus is on estimating the effects of particular conditions and our analyses reflect this goal; in comparing the conditions, however, the crucial issue was whether the credible intervals included 0 or not: If a credible interval includes 0, we refer to as an effect or result as “not credible”, if a credible interval excludes 0, we refer to an effect or result as “credible”. In the replication section, our data analysis corresponds to the analysis in the original study.

## Results

The results section is organized as follows: First, we present a qualitative replication of [9], again showing that doubtful and trustful respondents disagree about offering *£*10,000 when evaluating various clinical trial dimensions. Second, we offer a novel explanation on why respondents disagree on offering high or low pay by showing how payment can leak information about the riskiness of clinical trials. Third, we investigate the extent to which risks and coercion are linked to clinical trial evaluations (i.e., linking sections 1 and 2). Fourth, we test whether risk and coercion are credible determinants of repugnance for clinical trials more generally. Lastly, we explore individual differences in our sample; specifically, which characteristics increase the probability of a respondent being doubtful.

### Clinical trial evaluations (replication of Ambuehl et al., 2015)

Following [10], we categorized respondents into three types, based on how they rated the ethicality of different payment amounts in a side-by-side judgment at the end of the survey (*δ*_*rating*_ = *rating*_*£*10,000_ − *rating*_*£*1,000_). Trustful respondents rated a payment of *£*10,000 as strictly more ethically appropriate than a payment of *£*1,000 (*N* = 712, 50%, *δ*_*rating*_ = 1.84, *CI* = [1.77, 1.91]). Doubtful respondents rated a payment of *£*1,000 as strictly more ethical than a payment of *£*10,000 (*N* = 378, 27%, *δ*_*rating*_ = −2.24, *CI* = [−2.33, −2.14]). A subset of respondents rated both payment amounts equally (others, *N* = 338, 23%, *δ*_*rating*_ = 0.00). The proportions of each type in our British sample were highly similar to the proportions in the American sample reported in [9]—with a proportion of 44% trustful and 27% doubtful respondents. As in Ambuehl et al. (2015), our focal analyses pertain to doubtful versus trustful respondents.

**Fig 1:**
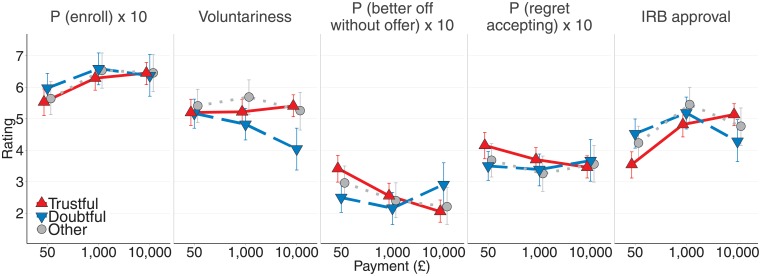
Responses of doubtful and trustful respondents for various payment amounts (between- respondents). Black triangles and circles represent sample means. Colored triangles and circles and error bars represent the means and the 95% highest density intervals of the posterior predictive distributions.

As in the original survey [9], all groups (trustful, doubtful, and others) evaluated clinical trials similarly as pay increased from *£*50 to *£*1,000 (see Fig 1): For instance, all respondents were more likely to give IRB approval for a trial offering *£*1,000 compared to one offering *£*50, likely because the trial was described as requiring 40 hours of commitment, for which *£*1,000 is fairer than *£*50. Fig 1 also shows that all respondents expected approximately the same number of people to enroll in a trial that offered *£*10,000 compared to one that offered *£*1,000 (all CIs across payment amounts and interactions between groups included 0). This differs from the original survey, where both groups expected more people to enroll for *£*10,000.

As in the original survey, however, offering *£*10,000 rather than *£*1,000 to clinical trial volunteers was evaluated differently by doubtful and trustful respondents with regard to “voluntariness,” “being better off without the offer,” “regret accepting” and—as a possible combination of the aforementioned concerns—“IRB approval.” Specifically, as Fig 1 shows, doubtful respondents considered the enrollment of a woman earning *£*1,500/month in the clinical trial as less voluntary when she was offered *£*10,000 (*b* = −0.97, *CI* = [−1.49, −0.44]). In addition, doubtful respondents said a woman earning *£*1,500/month would be better off had she not even seen the high offer (*b* = 1.26, *CI* = [0.26, 2.26]). Consistent with this, doubtful respondents also considered a woman earning *£*1,500/month to be more likely to regret accepting the offer (*b* = 0.52, *CI* = [0.04, 1.01]). Lastly, doubtful respondents on an IRB panel approved the study offering *£*1,000 more strongly than they approved the same study offering *£*10,000 (*b* = −1.22, *CI* = [−1.69, −0.77]; all models tested the effect of offering *£*10,000 over *£*1,000, using “trustful respondents” as a baseline; see column e in Table 1). All differences reported above were credible (the CIs excluded 0).

**Table 1:** Effects of increasing payment from £1,000 to £10,000 for seven dependent variables (1-7 in the rows).

Variable	(a) Intercept Trustful, £1,000	(b) £10,000	(c) Doubtful	(d) Other	(e) £10,000× Doubtful	(f) £10,000× Other
(1) P (enroll)	6.29 (5.99; 6.58)	0.16 (-0.24; 0.56)	0.30 (-0.19; 0.79)	-0.25 (-0.27; 0.76)	-0.39 (-1.13; 0.37)	-0.24 (-0.98; 0.49)
(2) Voluntariness	5.22 (5.01; 5.43)	0.18 (-0.10; 0.46)	**-0.40** **(-0.74; -0.06)**	**0.47** **(0.10; 0.84)**	**-0.97** **(-1.49; -0.44)**	**-0.61** **(-1.13; -0.09)**
(3) P (better off)	2.55 (2.16; 2.93)	-0.50 (-1.02; 0.03)	-0.39 (-1.10; 0.25)	-0.15 (-0.83; 0.54)	**1.26** **(0.26; 2.26)**	0.31 (-0.67; 1.29)
(4) P (regret accepting)	3.70 (3.51; 3.89)	-0.24 (-0.50; 0.02)	-0.31 (-0.62; 0.00)	**-0.44** **(-0.78; -0.11)**	**0.52** **(0.04; 1.01)**	**0.54** **(0.07; 1.02)**
(5) P (regret rejecting)	5.02 (4.85; 5.19)	**0.31** **(0.08; 0.55)**	0.11 (-0.18; 0.40)	-0.14 (-0.45; 0.16)	-0.41 (-0.85; 0.04)	0.01 (-0.42; 0.45)
(6) Personal approval	5.31 (5.13; 5.50)	0.23 (-0.03; 0.49)	0.16 (-0.15; 0.46)	0.33 (0.00; 0.65)	**-0.78** **(-1.26; -0.31)**	**-0.63** **(-1.10; -0.16)**
(7) IRB approval	4.82 (4.64; 5.00)	**0.32** **(0.08; 0.56)**	**0.37** **(0.07; 0.67)**	**0.64** **(0.32; 0.95)**	**-1.22** **(-1.69; -0.77)**	**-1.01** **(-1.46; -0.56)**
Obs. (wave 1)	76		64	176		
Obs. (wave 2)	302		274	536		
Obs. (total)	378		338	712		

Estimated coefficients in columns are main effects (a-d) and interaction effects between respondent types from multivariate fixed-effects regressions (variable ∼ payoff × respondent type); using £1,000 and “trustful” as a baseline (see the Intercept column, a). One regression per dependent variable. Credible effects in bold.

A robustness check that included gender as a predictor revealed that women were more critical of the clinical trial presented in the vignette: More so than men, they thought that a woman volunteering may regret accepting the offer, and they were less likely to approve of the trial personally or on an IRB panel. The differences between doubtful and trustful respondents persisted after controlling for gender (see Supplementary Material 2.3.).

### Inferred side effects

**Fig 2:**
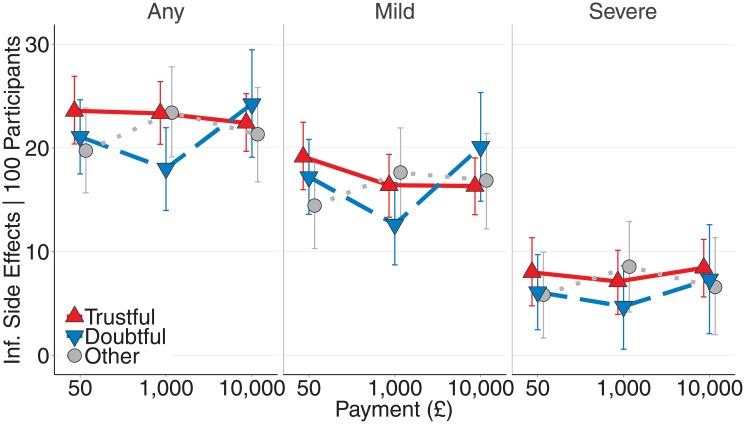
Estimated side effects for various payment amounts by doubtful, trustful, and other respondents (between-respondents). Black triangles and circles represent sample means. Colored triangles and circles and error bars represent the means and the 95% highest density intervals of the posterior predictive distributions.

Next, we investigated whether respondents perceived higher payment to be associated with higher risk—that is, a higher number of side effects. To test this possibility, we focused on the estimates for the first payment a respondent saw. We ran separate analyses for each type of side effect (any/mild/severe). There were no main effects of compensation amount on the estimated number of side effects (*£*50 versus *£*1,000, as well as *£*1,000 versus *£*10,000; all CIs included 0). However, recall that doubtful respondents found offering *£*10,000 to be less ethically appropriate than offering *£*1,000; whereas trustful respondents found offering *£*10,000 more ethically appropriate. A previously overlooked reason for this may be that doubtful respondents, unlike trustful respondents, took high payment amounts to be a cue for higher risks. As Fig 2 shows, increasing compensation from a very low amount (*£*50) to a moderate amount (*£*1,000) did not result in differential estimates of side effects for the two groups.

However, as in earlier analyses, individual differences emerged when a trial offered a very large compensation—*£*10,000, compared to *£*1,000. Specifically, doubtful respondents expected more side effects for trials that offered very large compensation compared to trustful respondents (*b*_*any*_ = 7.07, *CI* = [0.16, 13.87], *b*_*mild*_ = 7.52, *CI* = [2.05, 12.96], *b*_*severe*_ = 1.30, *CI* = [−2.63, 5.30], note the CI includes 0 for severe side effects), whereas trustful respondents’ inferred side effects were similar across payoff magnitudes (all CIs included 0; all models tested the effect of offering *£*10,000 over *£*1,000, using “trustful respondent” as a baseline). The differences in judgments for doubtful vs. trustful respondents were credible for any and mild side effects (the CIs excluded 0), see column e in Table 2). In sum, there was a positive relationship between the amount a clinical trial offers and its inferred riskiness for doubtful respondents. One qualification of this result is that extremely small payoffs (*£*50) did not reduce inferred risks compared to moderate amounts (*£*1,000)—only extremely large payoffs (*£*10,000) increased inferred risks compared to moderate amounts (*£*1,000).

**Table 2:** Effects of increasing payment from £1,000 to £10,000 for any, mild and severe side effects as dependent variables (1-3 in the rows).

Side effect type	(a) Intercept Trustful, £1,000	(b) £10,000	(c) Doubtful	(d) Other	(e) £10,000× Doubtful	(f) £10,000× Other
(1) Any	23.31 (20.63; 25.95)	-0.90 (-4.49; 2.68)	**-5.26** **(-9.60; -0.84)**	0.11 (-4.57; 4.73)	**7.07** **(0.16; 13.87)**	-1.23 (-7.82; 5.41)
(2) Mild	16.40 (14.26; 18.54)	-0.06 (-2.92; 2.82)	**-3.75** **(-7.28; -0.21)**	1.20 (-2.50; 4.94)	**7.52** **(2.05; 12.96)**	-0.65 (-6.02; 4.60)
(3) Severe	7.13 (5.57; 8.70)	1.30 (-0.81; 3.39)	-2.41 (-5.01; 0.19)	1.41 (-1.33; 4.14)	1.30 (-2.63; 5.30)	-3.27 (-7.19; 0.65)

Estimated coefficients in columns are main effects (a-d) and interaction effects between respondent types from multivariate fixed-effects regressions (side effect ∼ payoff × respondent type); using £1,000 and “trustful” as a baseline (see the Intercept column, a). One regression per side effect type. Credible effects in bold.

A robustness check that included gender as a predictor revealed that women generally estimated side effects to be higher compared to men. The differences between doubtful and trustful respondents persisted after controlling for gender (see Supplementary Material 3.3.).

### Inferred side effects and clinical trial evaluations

In considering why people consider high pay in clinical trials to be ethically inappropriate, we examined whether respondents’ ethical approval of the clinical trial was linked to their side effects estimates. Again, we focused on the first payment amount respondents saw. The number of participants expected to enroll in the study was unrelated to the estimated number of side effects (all CIs included 0): While the estimated number of side effects increased with increasing payment amounts from *£*1,000 to *£*10,000 (see Fig 2), *p (enroll)* did not (see Fig 1). It is plausible that inferred risks and high financial gain offset each other as respondents consider whether or not volunteers would enroll. However, a participant was considered to be better off without the offer if side effects were expected to be higher (*b*_*any*_ = 0.034, *CI* = [0.025, 0.043]). Consistent with this, respondents who estimated a higher number of side effects also thought that participants would be more likely to regret taking part in the study (*b*_*any*_ = 0.022, *CI* = [0.017, 0.026]). Lastly, higher risk assessments lowered respondents’ IRB approval of the clinical trial (*b*_*any*_ = −0.013, *CI* = [−0.017, −0.009], all modeled as main effects; note that coefficients are smaller for the side effects coefficients due to the side effects scale [0–100] as compared to the voluntariness scale [0–7]). All associations reported above were credible (the CIs excluded 0).

Importantly, the role of estimated side effects in evaluating the trial did not depend on whether a respondent was doubtful or trustful (clinical trial evaluations modeled in three-way interaction using response type [doubtful vs. trustful] × payment × estimated number of side effects as predictors; all CIs included 0). This is unsurprising since comparable interaction effects between these groups were present in clinical trial evaluations (see Fig 1) and the estimated number of side effects (see Fig 2). The results also held in other specifications of the model, for instance after controlling for the [doubtful vs. trustful] × payment [*£*50/*£*1,000/*£*10,000] interaction or the other side effect types as predictors [mild/severe] (see S4.1 analyses). These findings suggest that subjective risk estimates influence peoples’ approval of a given trial.

Earlier research suggested that these evaluations were also related to how coercive respondents found the clinical trial [9]. To study whether both inferred risks and coercion (i.e., lower voluntariness) have separable influences on clinical trial evaluations, we simultaneously entered voluntariness and side effects as predictors in a linear regression and found that both variables do explain unique variation in how respondents evaluated clinical trials. Participants were considered to be better off without seeing the offer when voluntariness was judged to be lower and inferred side effects were higher (*b*_*volun*._ = −0.3709, *CI* = [−0.4819, −0.2594], *b*_*risk*_ = 0.0229, *CI* = [0.0209, 0.0390]). Participants were judged to be more likely to regret accepting a trial less if voluntariness was judged to be lower and inferred side effects were higher (*b*_*volun*._ = −0.2612, *CI* = [−0.3118, −0.2107], *b*_*risk*_ = 0.0188, *CI* = [0.0146, 0.0229]). Moreover, IRB approval increased when voluntariness was judged to be higher and inferred side effects were lower (*b*_*volun*._ = 0.2800, *CI* = [0.2291, 0.3311], *b*_*risk*_ = −0.0098, *CI* = [−0.0139, −0.0057]; regression tables reported in S1 Supplementary materials 4.1). These associations were credible (the CIs excluded 0). We also explored the net effects payoffs had on IRB approval for doubtful and trustful respondents using a mediation approach: As expected, there was a smaller net effect of payoff on IRB approval for doubtful and not trustful respondents after controlling for estimated side effects and voluntariness (see S4.2 analyses). A similar pattern emerged in within-respondent analyses: Doubtful respondents relied on payoff information when assessing risks and voluntariness, and ultimately when determining IRB approval (see S4.3 analyses). Trustful respondents were less sensitive to changes in payoff magnitude.

### Repugnance

Our next goal was to characterize factors that shape repugnance. Although “repugnance is hard to predict” [8] (p. 42), there may be generalizeable factors that make some transactions more repugnant than others. It has not been assessed whether risk is one such factor. We did this here by using the repugnance measure developed for the second survey wave and linking it to respondents’ estimated number of side effects. That is, we examined whether the perceived risk of a clinical trial at least partially explains whether or not individuals find clinical trials repugnant. We hypothesized that respondents who considered clinical trials to be riskier may also find them more repugnant, or less ethically permissible in general (independent of payoff magnitude).

Indeed, higher repugnance was linked to a higher number of estimated side effects (*b*_*£*10,000_ = 0.0082, *CI* = [0.0058, 0.0106], estimates for “any” side effects, given a payoff of *£*10,000). This link was stable across side effect estimates for different payment amounts and in other specifications of the model (see S4.2). The link between side effects and repugnance was present for doubtful respondents (*b*_*£*10,000_ = 0.0082, CI = [0.0039, 0.0125) and trustful respondents (*b*_*£*10,000_ = 0.0048, *CI* = [0.0013, 0.0082]; main effects; comparable results for *£*1,000 and *£*50)—again, the link was more pronounced for doubtful respondents (comparison of *β* coefficients). Since coercion is another explanation for repugnance [9], we also tested whether voluntariness affected repugnance ratings in a regression using both side effects estimates and voluntariness as predictors. As Table 3 shows, clinical trials were considered less repugnant when they were judged to be more voluntary. This held in addition to the variance explained by the estimated number of side effects. We also included demographic variables in this analysis as, for instance, women may find clinical trials that recruit female volunteers more repugnant. As Table 3 shows, this was not the case—gender did not credibly predict repugnance. Instead, respondents who had thought about participating in clinical trials and/or were willing to take health risks, and had a higher income all considered the clinical trial to be less repugnant (Table 3). Being doubtful was not a credible predictor (CI includes 0).

**Table 3:** Judged repugnance predicted from a single multivariate model that included side effects, voluntariness, and demographic variables.

	Coefficient	95% credible interval
**Side effects**	**0.006**	**[0.003; 0.008]**
**Voluntariness**	**−0.160**	**[−0.193; −0.128]**
**Risk taking (health)**	**−0.032**	**[−0.059; −0.005]**
**Income**	**−0.070**	**[−0.134; −0.001]**
**Gender (male)**	−0.107	[− 0.217;0.003]
**Thought about participating**	**−0.252**	**[−0.500; −0.006]**
**Doubtful**	−0.107	[− 0.234;0.021]

The combined model reveals that a higher number of estimated side effects increases repugnance; lower voluntariness (i.e., higher coercion) has the opposite effect. Beyond these predictors, whether or not respondents had thought about participating themselves, their self-reported willingness to take health risks and income predicted additional, unique variance (credible intervals excluded 0). Being doubtful was not a credible predictor (credible interval includes 0). Credible differences in bold.

### Characteristics of doubtful respondents

Doubtful respondents were critical of offering *£*10,000 and seemed to make stronger payment-dependent inferences than trustful respondents. To better understand what differentiates between the two response types, we explored which characteristics increase the probability of a respondent being doubtful. Interestingly, respondents’ numeracy was positively associated with being doubtful (*b*_*numeracy*_ = 0.60, *CI* = [0.25, 0.94])—which may have led them to make stronger assumptions about a lack of “free lunches” in clinical trial markets. Moreover, respondents’ willingness to take health risks was weakly positively linked to being doubtful (*b*_*healthrisk*_ = 0.071, *CI* = [0.01, 0.13]), but not whether they had considered participating in medical trials themselves (*b*_*thought*(*participate*)_ = 0.14, *CI* = [−0.13, 0.40]). Demographic variables linked to being doubtful were income, age, and education (*b*_*log*(*income*)_ = 0.21, *CI* = [0.05, 0.39], *b*_*age*_ = 0.015, *CI* = [0.005, 0.025], *b*_*education*_ = 0.27, *CI* = [0.15, 0.39]), but not gender (*b*_*male*_ = −0.15, *CI* = [−0.41, 0.11]; all modeled as separate, main effects in a logistic regression predicting “doubtful”).

As Table 4 shows, in a logistic regression with all of these predictors added simultaneously, most of these results are robust. Only the income coefficient is not credible in this regression (*b*_*log*(*income*)_ = 0.14, *CI* = [−0.06, 0.35]). It is plausible that age capture some variability that is also captured by “Income”, as age and income are correlated (*b*_*age*_ = 0.004, *CI* = [0.001, 0.008]).

**Table 4:** Demographic characteristics of doubtful respondents.

	Coefficient	95% credible interval
**Numeracy**	**0.41**	**[0.02, 0.78]**
**Education**	**0.37**	**[0.22, 0.53]**
**Risk taking (health)**	**0.09**	**[0.02, 0.17]**
**Income**	0.14	[−0.06, 0.35]
**Age**	**0.02**	**[0.01, 0.04]**

The combined model reveals that doubtful respondents were more numerate, more educated, were more willing to take health risks, and were slightly older than trustful respondents (credible intervals excluded 0). Credible differences in bold.

## Discussion

More than 90% of healthy clinical trial volunteers say money is their main motivation for taking part in clinical trials [5, 6]. At the same time, high paying clinical trials are sometimes and by some people judged ethically inappropriate. We identified two reasons why high-paying clinical trials can prompt a sense of repugnance. First, consistent with earlier research, high payments can be seen as coercive [9]. The term “coercive” is adapted from the research we replicated—but it has been argued that a genuine offer of money (without a threat) cannot be coercive and that high monetary offers can only lead to undue influence [4]. Second, as we hypothesized, high pay can be used as a cue to the risk involved. Both reasons were not equally important to everyone. Specifically, doubtful respondents expected more people to experience side effects than people in the same trial with a smaller payoff, while trustful respondents inferred similar risks across payoff magnitudes. In contrast to a proposition by [9], doubtful respondents made this inference for *£*1,000 versus *£*10,000 payment schemes despite the trials being otherwise identical. Moreover, doubtful respondents considered coercion to be more problematic than trustful respondents (for *£*1,000 versus *£*10,000). Ultimately, as we have shown, such inferences can lead to lower IRB approvals, and volunteers are thought to be better off without being offered a high-paying clinical trial. Our findings do not support the conjecture that high pay impairs people’s ability to think carefully about the risks and benefits of a clinical trial [17]; nor do they support the idea that high pay conceals the risk involved in a clinical study [3, 16].

Prior research shows that people are usually aware that the high rewards they desire—in market transactions and many other domains in life—rarely occur as a windfall [25]. Consistent with this, some people seem to assume that there is no “free lunch” in clinical trial markets and infer that high payments compensate for high risk. However, such an assumption seems to be incorrect—or at least too simple—in the domain of clinical trials, given that pay is determined by myriad factors that play out differently across research institutes and studies [4]. One study finds that only a third of surveyed research institutes claimed to factor in risk when determining payment [26]. Moreover, since descriptions of clinical trials must disclose anticipated risks, payoff information will never be the only cue people can rely on to infer the possible risk involved in a clinical trial. For instance, in our vignette people were told that, “since no side effects occurred in animal studies, the institute’s experts consider it unlikely that they will occur in humans.”, and, “if side effects occur, they may range from […] nausea to […] migraines.” Overall, the fewer additional cues people have, the more they may focus on payoff information to infer probabilities [27]. More generally, people may have prior beliefs about the phase of the clinical trial and the medication in question. Thus, payment may be just one of several cues that could characterize the risk a study’s involves. This may help explain variability in earlier results [14, 15].

There were individual differences in that doubtful respondents were more critical of offering *£*10,000 than trustful respondents. An exploratory analysis revealed that a higher education, higher age and respondents’ willingness to take health risks increased the probability of a respondent being doubtful. Further research should target the extent to which a persons’ doubt or suspicion towards market transactions is stable across domains. For example, does being doubtful towards clinical trials mean that a person is also doubtful towards other, similar market transactions (e.g. those that commodify the human body in one way or another, such as surrogate motherhood, blood donation)—if the market transaction offers high pay? Indeed, some people have been found to trade off repugnance with market efficiency, while others take a strong moral perspective [28]. Conversely, it is also plausible that one and the same person judges the repugnance of markets differently in different situations—for instance based on the extent to which the person feels affected by the transaction, or the extent to which their culture approves the specific commodification.

Our experimental design has a few limitations. First, we investigated when clinical trials are considered repugnant by third parties. Therefore, our data do not speak to whether clinical trial volunteers themselves feel coerced to participate; nor the extent to which they consider high-paying trials to be riskier. From the perspective of a volunteer, risks in clinical trials are ideally minimized. They sign an informed consent that outlines the details of the study including potential risks, and they are free to leave at any point during the trial. Another limitation concerns the magnitude of the high payment and low payments (adopted from Ambuehl et al., 2015). Although clinical trials often offer much higher pay than minimum wage, a clinical trial payment of *£*10,000 for a 40 hour commitment may be unusually high, whereas *£*50 for a 40 hour commitment may be unusually low. Lastly, the vignette mentioned women as clinical trial volunteers, which may have raised concerns regarding gender inequality by both male and female respondents and thereby affected the true treatment effect. Additional research is needed investigate whether market transactions targeting the general population, as opposed to a particular subgroup, are viewed with a similar degree of repugnance.

Overall, our findings help to better understand *why* clinical trials are sometimes considered repugnant by those external to the transaction. Specifically, we showed that trials perceived to be riskier were considered to be more repugnant. Some repugnant markets are characterized by exposing sellers to risk (see [8], Table 1), but to our knowledge the role of subjective, inferred risk has been overlooked when studying feelings of repugnance. This link between risk and repugnance may generalize to other markets in which parties can be partially remunerated for the risk they take, such as surrogate motherhood. These findings have policy implications for both research institutes and governments. Currently, very high payments are perceived as compensation for higher risks by some. Although some research institutes do indeed compensate volunteers based on anticipated risks [13], an invariable general link between pay and risk in clinical trials is not in place, and high payments are often discouraged (e.g., to prevent undue influence). Policy makers need to be aware of the possibility that a payment-risk inference will be drawn. In response to this, it should be ascertained and communicated that clinical trial volunteers are paid for their time and inconvenience—not the risk they take—and that all measures are taken that minimize potential harm.

## Supporting information

S1 Supplementary materials Supplementary material for “When money talks”.

## References

[1] Nussbaum MC. “Whether from reason Or prejudice”: Taking money for bodily services. The Journal of Legal Studies. 1998;27(S2):693–723. 10.1086/468040

[2] AFP. Man who died in French drug trial had ‘unprecedented’ reaction, say experts. The Guardian. 2016. Retrieved from https://www.theguardian.com/science/2016/mar/07/french-drug-trial-man-dead-expert-report-unprecidented-reaction

[3] McNeill P. Paying people to participate in research: Why not? Bioethics. 1997;11(5):390–396. 10.1111/1467-8519.00079 11655118

[4] Largent EA, Lynch HF. Paying research participants: Regulatory uncertainty, conceptual confusion, and a path forward. Yale Journal of Health Policy, Law, and Ethics. 2017;17(1):61–141. 29249912 PMC5728432

[5] Bigorra J, Baños JE. Weight of financial reward in the decision by medical students and experienced healthy volunteers to participate in clinical trials. European Journal of Clinical Pharmacology. 1990;38(5):443–446. 10.1007/bf02336681 2379528

[6] van Gelderen CEM, Savelkoul TJF, van Dokkum W, Meulenbelt J. Motives and perception of healthy volunteers who participate in experiments. European Journal of Clinical Pharmacology. 1993;45(1):15–19. 10.1007/bf00315344 8405024

[7] Wilkinson M, Moore A. Inducement in Research. Bioethics. 1997;11(5):373–389. 10.1111/1467-8519.00078 11655117

[8] Roth AE. Repugnance as a Constraint on Markets. Journal of Economic Perspectives. 2007;21(3):37–58. 10.1257/jep.21.3.37

[9] Ambuehl S, Niederle M, Roth AE. More money, more problems? Can high pay be coercive and repugnant? American Economic Review. 2015;105(5):357–360. 10.1257/aer.p20151034

[10] Ambuehl S. An offer you can’t refuse? Incentives change what we believe. SSRN Electronic Journal. 2017.

[11] Fric C. Statutory minimum wages in the EU 2016.

[12] Pike ER. Recovering from research: A no-fault proposal to compensate injured research participants. American Journal of Law & Medicine. 2012;38(1):7–62. 10.1177/009885881203800101 22497093

[13] Dickert N, Emanuel E, Grady C. Paying research subjects: An analysis of current policies. Annals of Internal Medicine. 2002;136(5):368–373. 10.7326/0003-4819-136-5-200203050-00009 11874309

[14] Cryder CE, London AJ, Volpp KG, Loewenstein G. Informative inducement: Study payment as a signal of risk. Social Science and Medicine. 2010;70(3):455–464. 10.1016/j.socscimed.2009.10.047 19926187

[15] Mantzari E, Vogt F, Marteau TM. Does incentivising pill-taking’crowd out’ risk-information processing? Evidence from a web-based experiment. Social Science and Medicine. 2014;106:75–82. 10.1016/j.socscimed.2014.01.020 24534735 PMC3969102

[16] Bentley JP, Thacker PG. The influence of risk and monetary payment on the research participation decision making process. Journal of Medical Ethics. 2004;30(3):293–298. 10.1136/jme.2002.001594 15173366 PMC1733848

[17] Casarett D, Karlawish J, Asch DA. Paying hypertension research subjects: Fair compensation or undue inducement? Journal of General Internal Medicine. 2002;17(8):651–653. 10.1046/j.1525-1497.2002.11115.x PMC1495094 12213148

[18] Czarny M, Kass NE, Flexner C, Carson KA, Myers R, Fuchs E. Payment to healthy volunteers in clinical research: the research subject’s perspective. Clinical Pharmacology & Therapeutics. 2010;87(3):286–293. 10.1038/clpt.2009.222 20090675 PMC2946170

[19] National Minimum Wage. Retrieved from http://www.minimum-wage.co.uk/

[20] Cokely ET, Galesic M, Schulz E, Ghazal S, Garcia-Retamero R. Measuring risk literacy: The Berlin Numeracy Test. Judgment and Decision Making. 2012;7(1):25–47.

[21] Dohmen T, Falk A, Huffman D, Sunde U, Schupp J, Wagner GG. Individual Risk Attitudes: Measurement, Determinants and Behavioral Consequences. Journal of the European Economic Association. 2011;9(3):522–550. 10.1111/j.1542-4774.2011.01015.x

[22] Kruschke JK. Doing Bayesian data analysis: A tutorial with R, JAGS, and Stan, 2nd Ed New York, NY: Academic Press/Elsevier; 2014.

[23] RStanArm Version 2.9.0-4; 2016. Retrieved from cran.r-project.org/web/packages/rstanarm/.

[24] Gelman A, Rubin DB. Inference from iterative simulation using multiple sequences. Statistical Science. 1992;7(4):457–472. 10.1214/ss/1177011136

[25] Pleskac TJ, Hertwig R. Ecologically Rational Choice and the Structure of the Environment. Journal of Experimental Psychology: General. 2014;143(5):2000–2019. 10.1037/xge0000013 24979239

[26] Grady C. Payment of clinical research subjects. The Journal of Clinical Investigation. 2005;115(7):1681–1687. 10.1172/JCI25694 16007244 PMC1159153

[27] Leuker C, Pachur T, Hertwig R, Pleskac TJ. Exploiting risk–reward structures in decision making under uncertainty. Cognition. 2018;175:186–200. 10.1016/j.cognition.2018.02.019 29567432

[28] Elias JJ, Macis M, Elias JJ, Macis M, Lacetera N, Macis M. Efficiency-Morality Trade-Offs in Repugnant Transactions: A Choice Experiment. NBER Working Paper Series. 2016;(22632).

