# Supplementary material for: When money talks: Judging risk and coercion in high-paying clinical trials
Source: PLoS One. 2020 Jan 31;15(1):e0227898. doi: 10.1371/journal.pone.0227898 (PMC6994245; doi:10.1371/journal.pone.0227898)
Supplement: S1 Supplementary materials [file pone.0227898.s001.pdf]

Supplementary Material for "When money talks"

Christina Leuker[1]*, Lasare Samartzidis[1], Ralph Hertwig[1] and Timothy J. Pleskac[1,2]

[1]Max Planck Institute for Human Development, Berlin, Germany
[2]University of Kansas

November 18, 2019

Contents

1 Vignette **2**

2 Clinical trial evaluations **5**
2.1 Within-respondents results 5
2.2 Gender effects 5

3 Side effects **6**
3.1 Within-respondents results 6
3.2 Gender effects 6

4 Combined models **7**
4.1 Clinical trial evaluations (regressions) 7
4.2 Clinical trial evaluations (mediations) 8
4.3 Individual payoff sensitivity 9
4.4 Repugnance 10

5 Demographics **12**
5.1 Sample characteristics 12
5.2 Sample characteristics: Excluded respondents 13
5.3 Sample characteristics: Between conditions 13
5.4 Demographics & ethicality ratings 14

1 Vignette

Suppose that you are a member of an ethics committee, and you will have to decide whether or not to approve of the following study. Pay close attention. All the following questions will be based on this text.

The E.M.C.A. Medical Research Institute has developed a new vaccine to prevent infection with the Ebola virus. In rats and chimps the vaccine successfully prevents infection with the virus and causes no measurable side effects. The institute now seeks to enlist 100 female participants to investigate whether the vaccine causes side effects in women. This is important to know, as it will determine whether the vaccine can be given to female healthcare workers in regions affected by the disease.

Each of the 100 participants will be injected with the vaccine and then monitored in weekly intervals for two months. The total time required to participate if no side effects occur is about 40 hours. Participants will not be exposed to the virus; the study only tests for side effects of the vaccine. Since no side effects occurred in the animal studies, the institute's experts consider it unlikely that they will occur in humans. However, nobody knows for sure. This is why the experiment needs to be run. If unexpected side effects occur, they might range from very mild, such as a day of nausea, to very severe, such as persistent migraines. Side effects will be treated free of charge, if treating them is medically possible. An affected woman will not, however, receive treatment for any unrelated medical problems, and she will not receive any other compensation for suffering these side effects. The only compensation to any participant is the money paid to her when she agrees to take part in the study, before she is injected with the vaccine.

Study participation invitations will be posted throughout the city in which the institute is located. Invitations will be put up in both rich and poor neighborhoods. The institute will compensate each woman who participates with [£50/£1,000/£10,000] for the risk she takes and the time commitment required to participate in the study ($\sim$ 40 hours).

How many participants you would expect to have *any* side effects from taking part in the study? 0-100 [slider]

How many participants you would expect to have *mild* side effects from taking part in the study? 0-100 [slider]

How many participants you would expect to have *severe* side effects from taking part in the study? 0-100 [slider]

NEW PAGE

[Reminder of the introductory text]

Suppose you are a member of the ethics committee that has to approve the institute's study with [payment 1]. How would you decide? [7-point Likert scale with extremes labelled "definitely reject" and "definitely approve"]

How much do you personally approve of the institute's proposal to enlist and compensate study participants from both rich and poor neighborhoods in this way? [7-point Likert scale with extremes labelled "strongly disapprove" and "approve without reservation"]

NEW PAGE

A.S. is a woman who lives in a poor part of the city. For the past 20 years she has worked in various minimum-wage jobs. She currently earns £1,500 per month, which is barely enough for her to get by. A.S. encounters one of the study participation invitations that the institute has posted on bulletin boards in her neighborhood. A.S. considers signing up for this study. She is on the fence about whether or not to do so. She is afraid of possible unexpected serious side effects of the vaccine. But then again, she would be paid [as much as she earns in her job in a day / almost as much as she earns in her usual job in an entire month / more than six times as much as in her usual job in an entire month].

NEW PAGE

[Reminder of the text on A.S.]

Suppose that 10 women similar to A.S. see the institute's study participation invitation. How many of the 10 would be better off if the institute had never posted the study participation invitation? [0-10]

How many of the 10, do you think, will eventually participate in the study in exchange for [payment 1]? [0-10]

If A.S. decides to participate in the study for [payment 1], how would you describe her decision? [7-point Likert scale with extremes labelled "she was coerced" and "her decision was entirely voluntary"]

If A.S. decides to participate in the study, how likely is it that she will later regret her decision? [7-point Likert scale with extremes labelled "extremely unlikely" and "extremely likely"]

If A.S. decides NOT to participate in the study, how likely is it that she will later regret her decision? [Answer choices: 7-point Likert scale with extremes labelled "extremely unlikely" and "extremely likely"]

Researchers at the institute discuss offering payment [2, 3] for participation instead. (Each participant saw the same questions again for the other two payment amounts. These data were used for within-respondent analyses.)

For each of the following ways of compensating study participants, please indicate how ethically appropriate you think it is. Recall that the study test for effects of a vaccine, and although nobody expects such side effects to occur, if this were known, there would be no need to run a study. Recall that there is no special compensation if side effects occur. [7-point Likert scale with extremes labelled "completely unethical" and "completely ethical"]

- Do not pay money for participation
- Pay £50 for participation
- Pay £1,000 for participation
- Pay £10,000 for participation
- Pay everyone the amount of money for participation that he would earn at his job in 40 hours

NEW PAGE

[Reminder of the text on A.S.]

For your preferred way of compensating participants, please briefly explain why you think it is the most ethical way to do it. [box in which participants could fill in an essay]

NEW PAGE

Have you ever participated in a medical research study?

Have you ever thought about participating in a medical research study as a means to earn money?

Would you participate in the experiment about the Ebola vaccine described in this study for a payment of £50?

Would you participate in the experiment about the Ebola vaccine described in this study for a payment of £1,000?

Would you participate in the experiment about the Ebola vaccine described in this study for a payment of £10,000? [“yes”, “no”, “I do not know”. In the first two of the above five questions, the choice “prefer not to answer” was also available.]

To what extent do you agree/disagree with the following statements (about clinical trial markets) in general? A “clinical trial market” refers to research institutions being able to offer monetary incentives to prospective participants to take part in a clinical trial, who can freely agree or disagree to take part. [7-point Likert scale with extreme labelled “completely agree” and “completely disagree”]

- Clinical trial markets are deplorable.
- Clinical trial markets should be banned.
- Clinical trial markets should be tightly monitored by the government.
- Clinical trial markets are morally permissible.

Note. These are almost identical materials as in Ambuehl et al. (2015). The differences were as follows. We (1) added the side effects question, (2) removed “midwestern” from the city description and expressed the payment in *£* due to our primarily British sample, (3) in wave 2, added questions pertaining to how repugnant respondents considered clinical trial markets (see last question set), (4) asked respondents about their willingness to take risks and numeracy in addition to the other demographic variables (also see demographics table) and (5) appended an exploratory task in which respondents estimated their chances of loosing various hypothetical monetary amounts (see osf.io/5kewt/). We did not have predictions about how responses to this task are linked to our questions of interest here; the data will be analyzed and reported at a later point in time.

Ambuehl, S., Niederle, M., & Roth, A.E. (2015). More Money, More Problems? Can High Pay Be Coercive and Repugnant? *American Economic Review*, *105*(5), 357–360.

2 Clinical trial evaluations

2.1 Within-respondents results

Variable	(a) Intercept Trust., £1,000	(b) £10,000	(c) Doubtful	(d) Other	(e) £10,000× Doubtful	(f) £10,000× Other
(1) P (enroll)	6.33<br>(6.16; 6.50)	**1.72**<br>**(1.59; 1.84)**	**0.89**<br>**(0.61; 0.84)**	**0.60**<br>**(0.30; 0.91)**	**-0.32**<br>**(-0.54; -0.09)**	**-0.33**<br>**(-0.57; -0.10)**
(2) Volunt.	5.28<br>(5.14; 5.41)	-0.05<br>(-0.14; 0.04)	**-0.75**<br>**(-0.99; -0.14)**	0.14<br>(-0.10; 0.38)	**-0.98**<br>**(-1.14; -0.83)**	-0.13<br>(-0.29; 0.04)
(3) P (better off)	2.80<br>(2.56; 3.04)	**-0.55**<br>**(-1.76; -0.33)**	**-0.45**<br>**(-0.86; -0.33)**	-0.21<br>(-0.64; 0.21)	**0.75**<br>**(0.39; 1.12)**	**0.52**<br>**(0.14; 0.89)**
(4) P (regret acc.)	3.66<br>(3.54; 3.77)	**-0.62**<br>**(-0.71; -0.54)**	**-0.37**<br>**(-0.56; -0.17)**	**-0.36**<br>**(-0.56; -0.16)**	**0.44**<br>**(0.28; 0.60)**	**0.29**<br>**(0.13; 0.46)**
(5) P (regret rej.)	3.66<br>(3.55; 3.78)	**-0.62**<br>**(-0.72; -0.52)**	**-0.36**<br>**(-0.56; -0.16)**	**-0.36**<br>**(-0.56; -0.16)**	**0.44**<br>**(0.28; 0.61)**	**0.30**<br>**(0.13; 0.47)**
(6) Pers. app.	5.11<br>(4.99; 5.23)	**0.54**<br>**(0.44; 0.64)**	0.08<br>(-0.11; 0.28)	**0.25**<br>**(0.04; 0.46)**	**-1.52**<br>**(-1.69; -1.36)**	**-0.58**<br>**(-0.76; -0.41)**
(7) IRB app.	4.72<br>(4.60; 4.83)	**0.81**<br>**(0.06; 0.94)**	**0.21**<br>**(0.02; 0.40)**	**0.48**<br>**(0.28; 0.68)**	**-2.40**<br>**(-2.61; -2.19)**	**-1.11**<br>**(-1.32; -0.89)**

Table S1: Replication of Table 1 in the manuscript, but including all three payment amounts per respondent (within-respondent analyses). We accounted for random variation between respondents by including Response ID as a grouping factor. Estimated coefficients in columns are main effects (a-d) and interaction effects between respondent types from multivariate fixed-effects regressions (variable $\sim$ payoff $\times$ respondent type); using £1,000 and "trustful" as a baseline (see the Intercept column, a). One regression per dependent variable. Credible effects in bold.

2.2 Gender effects

As the vignette concerned female volunteers recruited for a clinical trial, female respondents may assess the clinical trial more critically than male respondents. We explored this possibility below.

Variable	Gender (female)	£10,000×Doubtful
P (enroll)	0.20 (-0.10; 0.50)	-0.42 (-1.17; 0.33)
Voluntariness	-0.18 (-0.39; 0.03)	**-0.94 (-1.46; -0.41)**
P (better off without)	0.04 (-0.37; 0.44)	**1.25 (0.28; 2.24)**
P (regret accepting)	**0.44 (0.25; 0.64)**	0.46 (-0.02; 0.93)
P (regret rejecting)	0.07 (-0.11; 0.25)	-0.41 (-0.85; 0.02)
Personal approval	**-0.24 (-0.43; -0.05)**	**-0.75 (-1.22; -0.27)**
IRB approval	**-0.27 (-0.45; -0.09)**	**-1.19 (-1.65; -0.73)**

Table S2: Replication of interaction effects in Table 1 in the manuscript, but including gender (between-respondent analyses). Women were more critical of the clinical trial regarding voluntariness, and compared to men they thought that a woman volunteering may regret accepting the offer. They also approved less of the clinical trial personally; and were less likely to give IRB approval. The interaction effects between payoff and being doubtful were robust.

3 Side effects

3.1 Within-respondents results

Side effect type	(a) Intercept Trustful, £1,000	(b) £10,000	(c) Doubtful	(d) Other	(e) £10,000× Doubtful	(f) £10,000× Other
(1) Any	23.01<br>(21.49; 24.56)	**1.61**<br>**(0.68; 2.53)**	-1.72<br>(-4.35; 0.91)	-2.47<br>(-5.23; 0.26)	1.57<br>(0.00; 3.14)	0.39<br>(-1.25; 2.03)
(2) Mild	16.84<br>(15.58; 18.11)	0.76<br>(-0.01; 1.56)	-0.88<br>(-3.08; 1.36)	-1.23<br>(-3.49; 1.03)	1.26<br>(-0.07; 2.57)	1.07<br>(-0.31; 2.47)
(3) Severe	8.20<br>(7.26; 9.14)	1.17<br>(0.54; 1.82)	**-1.78**<br>**(-3.38; -0.19)**	-1.04<br>(-2.69; 0.59)	**1.32**<br>**(0.24; 2.42)**	-0.32<br>(-1.47; 0.81)

Table S3: Replication of Table 2 in the manuscript, but including all three payment amounts per respondent (within-respondent analyses). We accounted for random variation between respondents by including Response ID as a grouping factor. Estimated coefficients in columns are main effects (a-d) and interaction effects between respondent types from multivariate fixed-effects regressions (side effect $\sim$ payoff $\times$ respondent type); using £1,000 and "trustful" as a baseline (see the Intercept column, a). One regression per side effect type. Credible effects in bold.

Note. We also tested for order effects. It is plausible that within-respondents effects would be larger when going from low to high payment amounts. Indeed, the within-respondent effects were mostly driven by respondents who were presented with £50 first, and the larger amounts later ($b_{Trustful}$ = 2.63, CI = $[1.04, 4.24]$, $b_{Doubtful}$ = 3.74, CI = $[1.89, 5.60]$, estimated number of any side effects when £50 was presented first). Because of this, we relied on between-respondent differences in our primary analyses.

3.2 Gender effects

As the vignette concerned female volunteers recruited for a clinical trial, female respondents may assess the clinical trial more critically than male respondents—and expect side effects to be higher. We explored this possibility below.

Side effect type	Gender (female)	£10,000×Doubtful
Any	**4.12 (1.39; 6.86)**	6.65 (-0.18; 13.41)
Mild	**3.30 (1.14; 5.46)**	**7.08 (1.59; 12.54)**
Severe	1.15 (-0.45; 2.73)	1.09 (-2.84; 5.06)

Table S4: Replication of interaction effects in Table 2 in the manuscript, but including gender (between-respondent analyses). Women generally estimated side effects to be higher compared to men (chosen as the reference group). Consistent with results reported in the manuscript, doubtful respondents still judged side effects to be higher in the £10,000 condition.

4 Combined models

4.1 Clinical trial evaluations (regressions)

Variables	(a) Side eff. any	(b) S&V: Side effects	(c) S&V: Voluntariness	(d) Side eff. mild	(e) Side eff. severe
P (enroll)	0.0017<br>(-0.0053; 0.0087)	0.0031<br>(-0.0040; 0.0102)	0.1190<br>(0.0290; 0.2048)	0.0000<br>(-0.0088; 0.0088)	0.0040<br>(-0.0084; 0.0163)
Voluntariness	–	–	–	–	–
P (better off without)	0.0339<br>(0.0248; 0.0429)	0.0299<br>(0.0209; 0.0390)	-0.3709<br>(-0.4819; -0.2594)	0.0448<br>(0.0334; 0.0563)	0.0548<br>(0.0388; 0.0709)
P (regret accepting)	0.0216<br>(0.0173; 0.0259)	0.0188<br>(0.0146; 0.0229)	-0.2612<br>(-0.3118; -0.2107)	0.0264<br>(0.0210; 0.0317)	0.0284<br>(0.0208; 0.0361)
P (regret rejecting)	-0.0068<br>(-0.0110; -0.0026)	-0.0063<br>(-0.0105; -0.0021)	0.0495<br>(-0.0021; 0.1017)	-0.0081<br>(-0.0132; -0.0029)	-0.0142<br>(-0.0214; -0.0069)
Personal approval	-0.0087<br>(-0.0132; -0.0042)	-0.0054<br>(-0.0097; -0.0011)	0.3051<br>(0.2522; 0.3581)	-0.0107<br>(-0.0163; -0.0051)	-0.0125<br>(-0.0201; -0.0048)
IRB approval	-0.0129<br>(-0.0173; -0.0086)	-0.0098<br>(-0.0139; -0.0057)	0.2800<br>(0.2291; 0.3311)	-0.0138<br>(-0.0193; -0.0084)	-0.0172<br>(-0.0147; -0.0096)
$\delta_{\ IRB\ approval}$*	-0.0190<br>(-0.0311; -0.0069)	-0.0180<br>(-0.0303; -0.0059)	0.0959<br>(-0.0561; 0.2472)	-0.0241<br>(-0.0391; -0.0090)	-0.0229<br>(-0.0444; -0.0012)
$\delta_{\ personal\ approval}$*	-0.0070<br>(-0.0161; 0.0023)	-0.0072<br>(-0.0166; 0.0021)	-0.0074<br>(-0.1233; 0.1078)	-0.0077<br>(-0.0190; 0.0037)	-0.0119<br>(-0.0279; 0.0043)
$\delta_{\ ethicality}$*	-0.0068<br>(-0.0084; 0.0048)	-0.0042<br>(-0.0158; 0.0074)	0.2634<br>(0.1187; 0.4070)	-0.0159<br>(-0.0307; -0.0009)	-0.0013<br>(-0.0216; 0.0190)

Table S5: Results for models linking clinical trial evaluations to respondents' estimated number of side effects. Columns a-c use the variable "any" side effects as the predictor. Column (a) shows the main effect; columns (b) and (c) show β coefficients from a model that included both side effects and voluntariness as predictors ("S&V", modeled as two main effects). Columns (d) and (e) show main effects using "mild" and "severe" side effects as predictors.

*same models as above, but including an interaction with payoff (using £1,000 as a baseline) — as the δs also refer to payoff–dependent differences in the evaluations.

Note. We also tested a number of other models, for instance controlling for doubtful/trustful × compensation amount, the interaction between side effects estimates and payment amount, or the three-way interaction doubtful/trustful × compensation amount × side effects estimates. The main effects of side effect estimates on the clinical trial evaluations was present in all models. Generally, and as can be seen from Table S5, a higher number of estimated side effects results in a less positive evaluation of a medical trial, and a higher degree of "voluntariness" for prospective participants results in a more positive evaluation of a medical trial.

4.2 Clinical trial evaluations (mediations)

We used a mediation approach suggested by MacKinnon et al. (2000) to show how payment information differentially affected IRB approval of doubtful and trustful respondents, given their differential estimates of side effects and voluntariness.

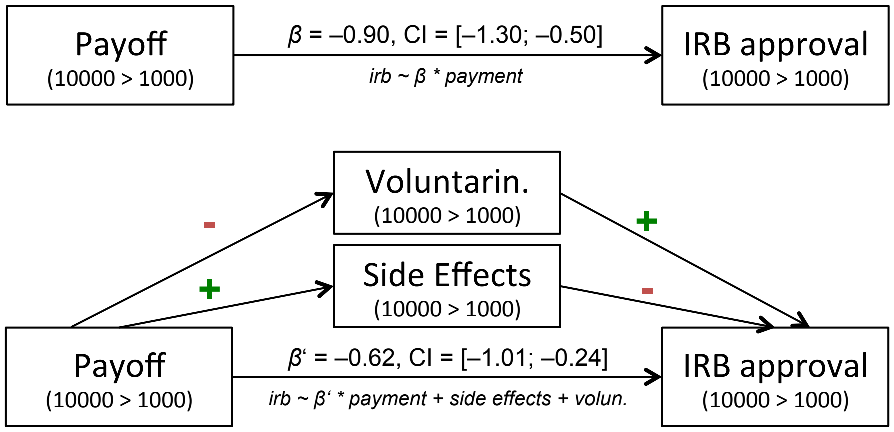

Figure S1: **Doubtful respondents.** Voluntariness and estimated side effects **lower** the role payoff plays in determining IRB approval (mediation effect: $\beta - \beta'$).

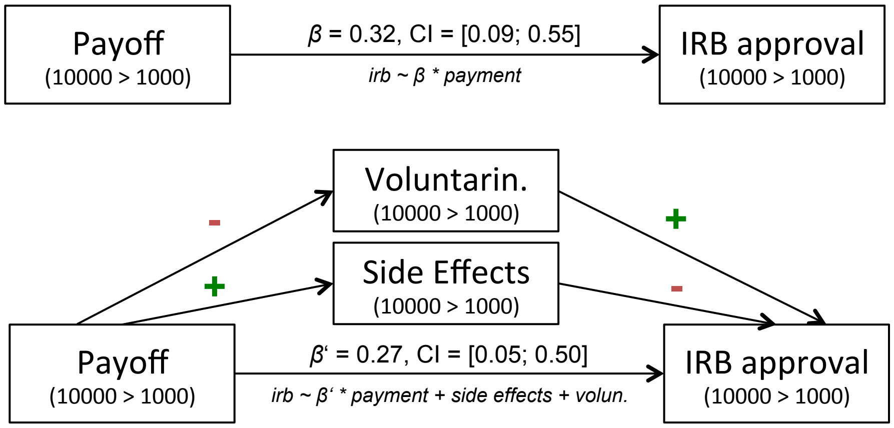

Figure S2: **Trustful respondents.** Voluntariness and estimated side effects **do not (strongly) affect** the role payoff plays in determining IRB approval substantially (mediation effect: $\beta - \beta'$).

MacKinnon, D. P., Krull, J. L., & Lockwood, C. M. (2000). Equivalence of the mediation, confounding, and suppression effect. *Prevention Science* (1), 173–181.

4.3 Individual payoff sensitivity

Our findings suggest that doubtful respondents (1) were more likely to utilize payment amount as a cue when estimating side effects; (2) also considered high payments to increase coercion (decrease voluntariness); and (3) evaluated high payments as less ethical (IRB approval). Is there a link between sensitivity to payoff information for IRB approval and side effects; and/or for IRB approval and voluntariness? In an exploratory analysis, we turned to within-respondent analyses for these dependent variables (i.e., using respondents' answers pertaining to each payment amount). For each respondent, we computed a "payoff sensitivity score" that measured how responses changed as clinical trials offered extremely high compensations. We did this for respondents' clinical trial evaluations (IRB approval: $\delta_{IRB} = IRB_{£10{,}000} - IRB_{£1{,}000}$), for their inferred number of any side effects [SE] ($\delta_{SE} = SE_{£10{,}000} - SE_{£1{,}000}$), and for voluntariness ($\delta_{volun.} = volun._{£10{,}000} - volun._{£1{,}000}$).

As Figure S3 shows, δ_{IRB} and δ_{SE} were inversely related for doubtful ($b = -0.018$, CI $= [-0.032, -0.004]$) but not for trustful respondents ($b = -0.006$, $CI = [-0.017, 0.004]$). In addition, δ_{IRB} and $\delta_{voluntariness}$ were positively related for both doubtful ($b = 0.246$, CI $= [0.110, 0.382]$), and trustful respondents ($b = 0.151$, CI $= [0.118, 0.243]$; $\delta_{voluntariness}$ not plotted). A comparison of β coefficients suggests that the link was stronger for doubtful respondents, again suggesting a stronger reliance on payoff information when evaluating clinical trials.

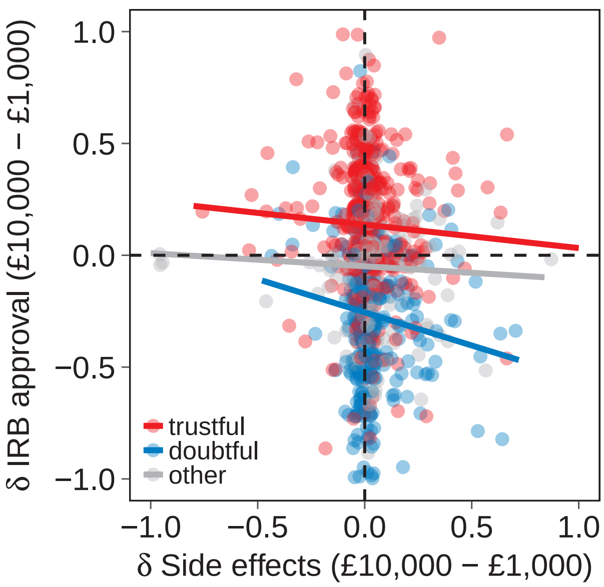

Figure S3: Relation between payoff sensitivity in the estimated number of side effects (normalized δ_{SE}) and payoff sensitivity in IRB approval ratings (normalized δ_{IRB}). A payoff-dependent increase in estimated side effects was linked to lower IRB approval rates for doubtful, but not trustful respondents (within-respondents). Each dot represents one participant. Note: Although the relationship is statistically reliable, as the density of the datapoints suggests, most respondents did not infer a different amount of side-effects—which is sensible given they saw the same vignette (just with a different payoff) repeatedly.

4.4 Repugnance

	(a) Side eff.	(b) S&V: Side eff.	(c) S&V: Volunt.	(d) Side eff. (£50)	(e) Side eff. (£1000)
**Repugnance**	0.0082<br>(0.0058; 0.0106)	0.0065<br>(0.0042; 0.0088)	-0.1489<br>(-0.1803; -0.1172)	0.0089<br>(0.0062; 0.0116)	0.0090<br>(0.0063; 0.0116)

Table S6: Columns a-c used estimates of "any" side effects for £10,000 as predictors. Column (a) shows the main effect; columns (b) and (c) show β coefficients from a model that included both side effects and voluntariness as predictors ("S&V", modeled as two main effects). Columns (d) and (e) show main effects using side effects estimates for £50 and £1,000.

	(a) Side eff. Doubtful	(b) Side eff. Other	(c) Side eff. Trustful
**Repugnance**	0.0082<br>(0.0039; 0.0125)	0.0142<br>(0.0092; 0.0194)	0.0048<br>(0.0013; 0.0082)

Table S7: Side effects. Main effects for each type. Each respondent is only entered in the regression once, with their estimate for "any' side effects given the £10,000 trial.

	(a) Volunt. Doubtful	(b) Volunt. Other	(c) Volunt. Trustful
**Repugnance**	-0.1240<br>(-0.1805; -0.0675)	-0.2386<br>(-0.3073; -0.1705)	-0.1531<br>(-0.1998; -0.1060)

Table S8: Voluntariness. Main effects for each type. Each respondent is only entered in the regression once, with their estimate for "any' side effects given the £10,000 trial.

	**Repugnance**
**Side eff.**	0.0063<br>(0.0039; 0.0087)
**Voluntariness**	-0.1606<br>(-0.1934; -0.1281)
**Risk (health)**	-0.0328<br>(-0.0598; -0.0058)
**Income**	-0.0701<br>(-0.1349; -0.0018)
**Gender (male)**	-0.1072<br>(-0.2172; 0.0033)
**Thought about participating**	-0.2524<br>(-0.5000; -0.0069)
**Doubtful**	-0.1071<br>(-0.2340; 0.0214)

Table S9: Repugnance predicted from side effects, voluntariness, and demographic variables (also see S5 for main effects). As before, the combined model reveals that a higher number of estimated side effects increases repugnance; lower voluntariness (i.e., higher coercion) has the opposite effect. Beyond these predictors, only whether or not respondents had thought about participating themselves and willingness to take health risks predicted additional, unique variance. Being doubtful was not a reliable predictor (CI includes 0).

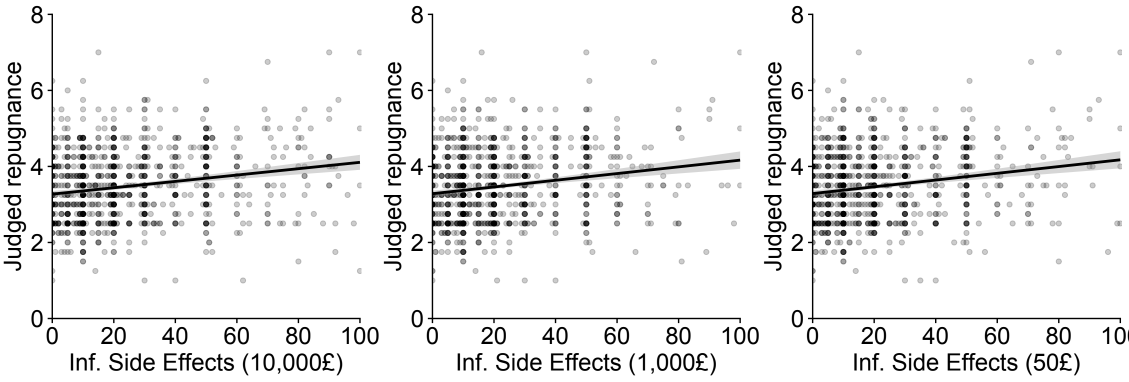

Figure S4: The link between risk (the estimated number of side effects) and repugnance held irrespective of the response type (doubtful/trustful/other).

5 Demographics

5.1 Sample characteristics

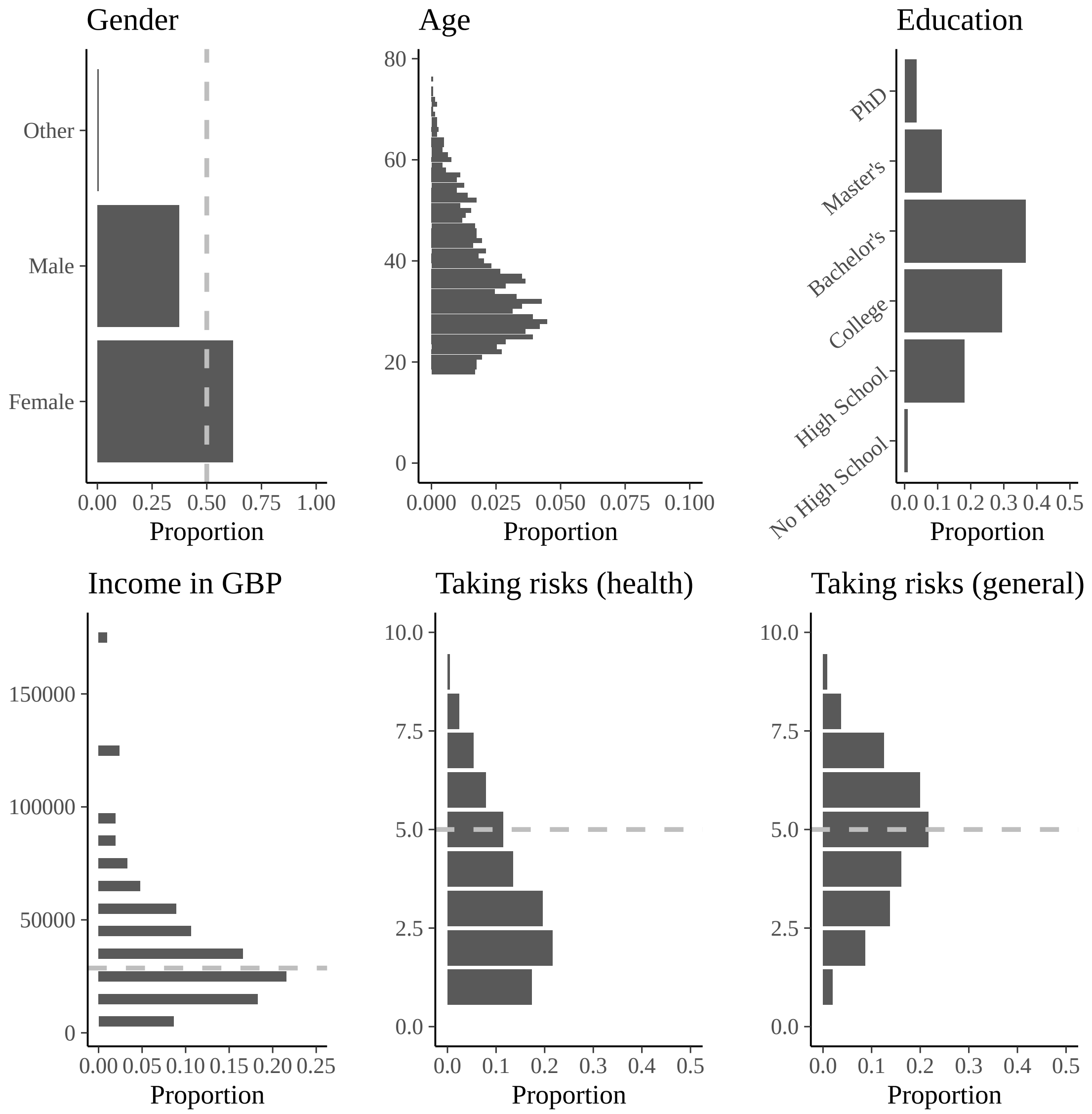

Figure S5: Characteristics of current sample. The sample consisted of a slightly higher proportion of women than men; and was slightly skewed toward younger age groups. The median income was roughly equivalent to the median income in the UK (dashed line). Our sample was risk averse toward taking health risks (a potential consequence of asking questions about clinical trials prior asking about health risks), and risk neutral toward taking general risks.

5.2 Sample characteristics: Excluded respondents

We assessed whether there were any systematicities in demographics for participants who were excluded (due to failed attention checks) vs. included. The analysis below predicts the probability of being excluded based on various demographic characteristics (Gender, Income, Education, Age). Baseline for Education: Advanced Graduate Work or PhD. Baseline for Gender: Female. All credible intervals included 0; suggesting little systematicity in exclusions.

	**Coefficient**	**95% credible interval**
Intercept	3.75	(1.02; 6.57)
Gender: Male	-0.19	(-0.58; 0.20)
Gender: Other	-0.59	(-2.80; 2.46)
log (Income)	-0.16	(-0.40; 0.08)
Education: Bachelors Degree	0.82	(-0.27; 1.77)
Education: Did not complete High School	-0.79	(-2.40; 0.86)
Education: High School	-0.39	(-1.47; 0.51)
Education: Masters Degree	0.21	(-0.94; 1.19)
Education: Some College	-0.09	(-1.14; 0.83)
Age	0.01	(-0.01; 0.02)

5.3 Sample characteristics: Between conditions

We assessed whether there were any systematicities in demographics for participants who were assigned to the £10,000 condition (compared to the £1,000 condition). The analysis below predicts the probability of being assigned to the £10,000 (high payoff) condition based on various demographic characteristics (Gender, Income, Education, Age). Baseline for Education: Advanced Graduate Work or PhD. Baseline for Gender: Female. All credible intervals included 0; suggesting that the random assignment worked as intended.

	**Coefficient**	**95% credible interval**
Intercept	-0.21	(-2.35; 1.86)
Gender: Male	-0.14	(-0.42; 0.13)
Gender: Other	-1.08	(-4.35; 1.54)
log (Income)	0.01	(-0.17; 0.19)
Education: Bachelors Degree	0.20	(-0.55; 0.98)
Education: Did not complete High School	0.60	(-0.90; 2.17)
Education: High School	-0.11	(-0.88; 0.70)
Education: Masters Degree	0.32	(-0.48; 1.15)
Education: Some College	0.05	(-0.71; 0.84)
Age	0.00	(-0.01; 0.01)

5.4 Demographics & ethicality ratings

**Variables**	$\boldsymbol{\delta}$ ***IRB approval***	$\boldsymbol{\delta}$ ***personal approval***	$\boldsymbol{\delta}$ ***ethicality***	**Repugnance**
Income	-0.29 (-0.42; -0.15)	-0.07 (-0.18; 0.03)	-0.20 (-0.34; -0.07)	-0.09 (-0.16; -0.01)
Education	-0.09 (-0.19; 0.00)	-0.07 (-0.09; 0.00)	-0.22 (-0.32; -0.13)	-0.06 (-0.11; -0.01)
Gender (male)	-0.16 (-0.36; 0.05)	-0.01 (-0.17; 0.05)	-0.01 (-0.20; 0.22)	-0.20 (-0.32; -0.08)
Age	-0.020 (-0.029; -0.012)	-0.004 (-0.010; 0.003)	-0.012 (-0.021; -0.004)	-0.006 (-0.010; -0.001)
Numeracy	-0.25 (-0.53; 0.04)	-0.12 (-0.33; 0.10)	-0.50 (-0.78; -0.22)	-0.15 (-0.29; 0.02)
Risk-taking (health)	0.00 (-0.05; 0.05)	-0.02 (-0.02; 0.02)	-0.05 (-0.08; -0.02)	-0.05 (-0.08; -0.02)
Risk-taking (general)*	0.05 (-0.02; 0.11)	0.01 (-0.04; 0.06)	0.08 (0.01; 0.14)	-0.01 (-0.04; 0.02)
Thought about participating	-0.06 (-0.28; 0.15)	-0.11 (-0.27; 0.05)	-0.10 (-0.32; 0.11)	-0.26 (-0.38; -0.15)
$\delta_{\ IRB\ approval}$				
$\delta_{\ personal\ approval}$	0.75 (0.70; 0.81)			
$\delta_{\ ethicality}$	0.57 (0.53; 0.61)	0.36 (0.33; 0.40)		
Repugnance*	0.119 (-0.005; 0.244)	0.091 (-0.004; 0.185)	0.080 (-0.043; 0.203)	

Table S10: Demographic variables and relationship to measures of interest. δs are the differences in respondents' evaluations of the clinical trial offering £10,000 vs. £1,000 ($\delta = Rating_{£10,000} - Rating_{£1,000}$). All coefficients are reported with their 95% credible intervals.

*only collected in wave 2

Note. As in the original survey, we find that personal approval and IRB approval are highly correlated ($\beta = .76$). Moreover, IRB approval and ethicality are correlated, but to a lesser extent ($\beta = .36$). The distinction between doubters and trustful respondents is based on $\delta_{\ ethicality}$.
